# Challenges and Prospects for Integrating the Assessment of Health Impacts in the Licensing Process of Large Capital Project in Brazil

**DOI:** 10.15171/ijhpm.2018.58

**Published:** 2018-06-30

**Authors:** Sandra de Souza Hacon, André Reynaldo Santos Périssé, Jean Simos, Nicola Luca Cantoreggi, Mirko Severin Winkler

**Affiliations:** ^1^Escola Nacional de Saúde Pública Sérgio Arouca (Ensp), Fundação Oswaldo Cruz (Fiocruz), Rio de Janeiro, Brazil.; ^2^Institute of Global Health, University of Geneva, Genève, Switzerland.; ^3^Swiss Tropical and Public Health Institute, Basel, Switzerland.; ^4^University of Basel, Basel, Switzerland.

**Keywords:** Health Impact Assessment, Health Policy, Large Capital Projects, Brazil

## Abstract

Brazil was one of the first countries in Latin America to institutionalize a National Environmental Policy in 1981, including the environmental impact assessment (EIA) process of economic activities with anticipated impacts on the environment. Today, EIA practice in Brazil comes with a number of limitations: it is constrained by its environmental advocacy role; application is strongly oriented towards large capital projects; and social responsibility considerations are only partially included. Consequently, EIA studies mainly address issues connected to localised and direct environmental impacts, largely ignoring any socio-economic and health impacts. This perspective paper highlights limitations of current EIA practice in Brazil with a focus on health considerations in impact assessment. While recognizing the positive impact to municipalities where large capital projects are being developed and operated, adverse impacts on health are a reality with measurable evidence in Brazil. Therefore, we argue that specificities on how to systematically assess and monitor potential health impacts cannot remain invisible in the Brazilian legislation, as currently seen in the reformulation of the licensing process in the country. The process of better integrating the assessment of health impacts in the licensing process of large capital project in Brazil must, however, not be based on the imposition of an external model but should be promoted by internal stakeholders from the environmental and health sector, incorporating the experiences gained in various case studies from all over the country.

## Introduction

Over the last decade, the quantity and magnitude of large capital projects – in particularly infrastructure developments such as improvement in urban mobility, mining, oil, gas and hydropower projects, as well as large-scale agribusinesses – have increased considerably in Brazil.^1^ Aiming at accelerating economic growth, the federal government launched the “Programa de Aceleração do Crescimento” (Growth Acceleration Program, PAC) in 2007. With investments of approximately US$300 billion, the PAC was meant to increase employment and improve living conditions of the Brazilian population by encouraging private investment, increasing public investment in infrastructure developments, and removing obstacles to growth. In the second phase of the PAC (PAC2), investments of US$580 billion were forecast for the period 2011-2014.^2^ In the state of Rio de Janeiro alone, 1588 projects were planned, including 45 in the transportation sector (eg, airports and highways) and 75 in the energy sector (eg, hydropower, oil and gas). Some prominent examples of large capital project that were developed under the PAC2 include: (*i*) the North-South railroad in the north-east region; (*ii*) the São Francisco river transposition; (*iii*) the Jirau, Santo Antônio and Belo Monte hydroelectric plants; and (*iv*) the Rio de Janeiro Petrochemical Complex.

Large capital projects in Brazil have great potential to promote economic growth and accelerate the development of the country. On the other hand, the various potential positive effects of the PAC and other large infrastructure developments are opposed by potential adverse impacts on the environment, society and health.^3^ In this perspective paper, we reflect on the licensing process of large capital projects in Brazil, with emphasis on existing barriers and future opportunities for integrating human health in the existing environmental impact assessment (EIA) legislation.

## The Evolution of Environmental Protection in Brazil

In the 1970s and 1980s, industrial activity in Brazil accelerated the extraction of natural resources, resulting in adverse impacts not only on the environment but also on health determinants of populations in the affected areas.^4,5^ Indeed, exploitation and destruction of natural areas such as “Sete Quedas” on the Iguaçu River, the construction of petrochemical plants in areas of aquifers needed for public water supply, deforestation and the advancement of logging and mining caused contamination of rivers, soils and the atmosphere, and degradation of coastal and interior areas through environmental pollution and population growth. Consequently, the so called “development projects” generated civil society reactions in Brazil and abroad, culminating in the enactment of the National Environmental Policy Act (law 6.938 of 31 August 1981; “Política Nacional do Meio Ambiente” - NEPA). In the final NEPA document, EIA was a mandatory requirement to be applied in the planning and decision-making process of capital projects with potential to generate pollution.

Following the principles established by the NEPA, legal instruments were formulated, such as the National Environment Council’s (CONAMA) Resolution No. 1, dating back to January 23, 1986, which introduced specific EIA regulations, setting the basic components of the Brazilian EIA system as defined within the legislation.^6,7^ As an instrument of policy and management, EIA aims to enable the use of natural and economic resources, promoting a comprehensive and in-depth *ex-ante* assessment, discussion and impartial analysis of possible positive and negative social-environmental impacts of large capital projects. In Brazil, this environmental licensing process includes three levels of licenses: (*i*) early license; (*ii*) installation license; and (*iii*) operation license. This licencing process is informed by the establishment of public hearings.

## Health in Environmental Impact Assessment

Historically, large capital projects have led to a massive migratory flow of a predominantly young and male population during the construction phase, with no prospect of future occupation.^8,9^ Several examples show that large capital projects lead to significant real estate speculation with local land price inflation, which occurs even before the beginning of construction work. This affects the entire local commercial sector and local communities, mainly in relation to food and drug prices, as well as the prices of residence rentals that tend to increase with the arrival of temporary inhabitants. Displacement and resettlement are also important project-induced effects that can negatively affect individuals’ health.^10^ The great temporary nature of the workers’ population, who in some cases come with their families, has been related to several health events such as increased violence, car accidents, sexually transmitted diseases, and communicable diseases such as tuberculosis, HIV and Hansen’s disease.^11-13^

Although the CONAMA Resolution defines that environmental impact is any change in the environment that directly or indirectly affects the health, safety and well-being of the population, large capital projects have often been developed without specific consideration of health and well-being impacts in affected communities. Indeed, an analysis of environmental reports of 21 national oil production enterprises, licensed between January 1, 2004 and October 31, 2009, found no evidence of the incorporation of health aspects in the large majority of EIAs.^14^ This may be explained by the fact that the first CONAMA Resolution provided no methodological or technical specificities on how health considerations should be included in the EIA. It was only in 2001 when health was more explicitly mentioned in a CONAMA Resolution (No. 286/2001), by inserting the request that specific studies on malaria are carried out in areas where the disease is endemic in order to avoid the increase of morbidity and mortality due to communicable diseases.^6^ It is also noteworthy that from 2009 onwards, CONAMA Resolution 420 established the procedures for assessing human health risks as a mandatory activity for contaminated areas and decommissioned projects. The proposed risk assessment approach is used in the evaluation of economic activities with the potential for chemical contamination, renewal of the licensing process, accidents and disasters with chemical substances or hazardous waste disposal.^6^

## Health Impact Assessment in Brazil

The World Health Organization (WHO) defines health impact assessment (HIA) as a “combination of procedures, methods and tools by which a policy, programme or project may be judged as to its potential effects on the health of a population, and the distribution of those effects within the population.”^15^ HIA was developed from the observation that in several countries EIA generally does not assess in-depth potential impacts of the enterprises on the health of the population in their areas of influence.^16,17^ As for EIA, HIA studies should be carried out during the planning stage of policies, programs and projects.^18^ In addition to the identification of measures for minimising adverse health impacts while maximising health opportunities associated with large capital projects, HIA also incorporates monitoring and evaluation of health impacts in affected populations over the course of project implementation.^13^

Since the early 21st century, the Brazilian Ministry of Health (BMoH) has been discussing the need to specifically assess and evaluate potential health impacts of large capital projects, linking them to the licensing process as it is being prescribed under the Brazilian EIA legislation. In 2014, the Ministry of Health published the first technical document on HIA, describing the approach and proposing the integration of health in the process of environmental licensing.^19^ Complementary to the promotion of HIA of large capital projects by the BMoH, researchers from the “Fundação Oswaldo Cruz” (Fiocruz) have initiated an HIA capacity building effort. In four 1-week training courses implemented in the years 2015-2017, almost 200 individuals from a broad set of institutions and sectors from all over the country received basic training on “HIA of large capital projects.”

## Obstacles to Implementation of Health Impact Assessment in Brazil

The Samarco mining dam disaster in the district of Mariana, state of Minas Gerais, directly and indirectly affected about 1.2 million people and demonstrated that the enforcement of existing laws and regulations in Brazil by public authorities is fragile.^20^ The vulnerability of the environmental monitoring system in Brazil, whether by federal, state or local authorities, and the lack of organisation and demands by the civil society for monitoring environmental problems, directly contribute to events like those in Mariana and others tragedies in Brazil. All environmental disasters, accidents and tragedies have direct and indirect health consequences, especially for the most vulnerable groups living in the areas of influence of large capital projects, usually the poorest. Some of the obstacles to the establishment of HIA in Brazil include (*i*) the demobilization of technical groups within environmental agencies, health and environmental departments; (*ii*) the technical difficulties of the Agency for Law Enforcement and Prosecution of Crimes (“Ministério Público”); (*iii*) a lack of training programs for updating and evaluating professionals from Brazilian environmental and health agencies; and (*iv*) consequently, the lack of commitment of environmental managers.^21-23^ Furthermore, in the area of health, there is a lack of a common language regarding the health impacts generated by major projects, as well as the integration of the health and environmental areas into associated proactive and retrospective measures. Politically, the authoritarian character of the federal and state governments, privileging large economic groups in the country and inducing changes in environmental legislation, has weakened the role of regulatory agencies for the national environment policy, leading to their disinterest in the country’s democratic management of social-environmental problems. Regarding the implementation of HIA in projects, programs and policies, in the current political scenario there is no commitment by the federal or state governments to advance the procedures for implementing HIA. Many entrepreneurs and environmental professionals envision HIA as a cost to industry rather than as a health benefit to the exposed population and a long-term economy for industry and government. The impact of the Mariana disaster on the environment and health is one of the examples of the lack of commitment by the state and federal governments towards health impacts of large mining projects. Such impacts are long-lasting and similar future disasters must be avoided. In this sense, the following are crucial measures for the success of the implementation of any large capital projects and reduction of their potential negative health impacts:

- objective and comprehensive definition and screening of capital projects with potential to cause great environmental and health impact in affected populations;
- technical experts that are capable to conduct comprehensive HIA;
- adequate planning of large capital projects, including public participation;
- detailed studies on the health of population groups residing in affected areas; and
- technical staff at the level of public regulatory agencies that have the skills and experience to review and evaluate HIA documentations and are able to carry out inspections in project areas.

Contrary to these assumptions, and at an untimely moment due to discussions about the causes and culpability in the rupture of the Samarco mining dam, the Brazilian National Congress and the CONAMA are discussing changes in environmental legislation with the basic objective of making environmental licensing of large capital projects more agile, flexible and fragile. Similar processes of environmental health status worsening in the name of struggling against economic or financial crisis are observed in other countries.^24^ The action fronts are varied, ranging from the resumption and adaptation of an old Bill in the Federal Senate that modifies the National Environmental Policy (PLS no. 654/2015) to a proposal of discussions towards a new CONAMA resolution with guidelines for environmental licensing in which the mere presentation of an environmental impact study leads to authorization for the continuation of the project.^6,25^ Simplification and streamlining EIA process has been a recurring concern among environmental professionals, especially those who work in the licensing process and researchers. On the other hand, recent regulatory responses to such concerns are being perceived as a threat to EIA effectiveness and environmental protection.^26^

Some laws under discussion modify the logic of the three-phase licensing in force today, facilitating the licensing of large capital projects vaguely defined by law. There are also proposals for a large reduction in the deadlines for licensing, even in projects that require three-phase licensing, and virtual extinction of the public consultation. It has also been debated about the creation of the concept of strategic projects which would be subject to a slackened environmental licensing process, the creation of the Unified Licensing Office to issue licenses placing all the institutions involved in the licensing process into a single body, a 6-month maximum deadline for issuing environmental licenses, and a reduction of the protection in conservation areas allowing mining, agribusiness and other enterprises in such areas. Overall, discussions are centred in facilitating and shortening the environmental process.

## Conclusion

Currently, there is no legal framework or well-established conceptual references for adopting HIA in Brazil and the discussion about its integration within the laws and regulations body. In this sense, it is necessary that efforts to insert this instrument are less based on the imposition of an external model and much more on the promotion of internal discussions and Brazilian case study experiences, especially within the environmental and health sectors. Researchers can contribute by developing case studies that present evidence of health impacts about large capital projects. The health sector has yet to show that HIA complements effectively the EIA of large capital projects, with a positive contribution to the decision makers. HIA should be seen as a process of strengthening health promotion rather than competing with the environmental sector.^25^ The main opportunity for integrating health in EIA legislation is by introducing such proposal in the current discussions about changes into the existing EIA system. The ongoing revision in the EIA regulation can be a great opportunity to propose changes to the environmental legislation that could improve health assessment. On the other hand, simplification, flexibility and cost are the main concerns and threat to EIA effectiveness. Therefore, we understand that HIA needs to be discussed and present evidences and benefits in terms of positive impacts to politics, business organizations and politic parties.

In this period of turbulence in the executive, legislative, and judiciary branches of the government, a decisive factor in advancing the HIA process could be based on an improved understanding between local, state and federal environmental and health agencies, such as the Brazilian Institute of the Environment and Renewable Natural Resources (IBAMA), municipal, state and federal health departments, agencies for law enforcement and prosecution of crimes, and the Ministry of the Environment. Exchanges should include the discussion of evidence on health impacts in large capital projects, the advantages and disadvantages of including HIA in the licensing process, and the need for capacity building in these institutions to form a technical staff capable of informally initiating a discussion about HIA with some NGOs with international visibility and some actors in the private sector with the National Development Bank (BNDES). This should be done to gage BNDES’s receptivity in relation to the advance of a political proposal for HIA in the current discussion about licensing processes underway by Lawmakers.

## Acknowledgments

This work was made possible by the support and funding received from the ‘Science without Borders’ (Ciências sem Fronteira) programme of the Brazilian government (CAPES- process number 88887.094769/2015-00).

## Ethical issues

Not applicable.

## Competing interests

Authors declare that they have no competing interests.

## Authors’ contributions

SSH, AP, and MSW conceptualized and drafted the manuscript. JS and NLC reviewed the manuscript. SSH, AP, MSW, JS, and NLC finalized the manuscript.

## Authors’ affiliations

^1^Escola Nacional de Saúde Pública Sérgio Arouca (Ensp), Fundação Oswaldo Cruz (Fiocruz), Rio de Janeiro, Brazil. ^2^Institute of Global Health, University of Geneva, Genève, Switzerland. ^3^Swiss Tropical and Public Health Institute, Basel, Switzerland. ^4^University of Basel, Basel, Switzerland.
